# Analysis of the complete mitochondrial genome sequence of *Palinura homarus*

**DOI:** 10.1080/23802359.2017.1280703

**Published:** 2017-02-02

**Authors:** Bao-hua Xiao, Wuchai Zhang, Wei Yao, Chu-wu Liu, Li Liu

**Affiliations:** a Key Laboratory of Aquaculture in South China Sea for Aquatic Economic Animal, College of Fisheries, Guangdong Ocean University, Zhanjiang, China;; b Shenzhen Research Institute of Guangdong Ocean University, Shenzhen, China

**Keywords:** *Palinura homarus*, mitogenome, coded protein, tRNA

## Abstract

The complete mitochondrial genome sequence of *Palinura homarus* was obtained using PCR amplification and walking sequencing (GenBank accession no. JN_542716). The complete mitochondrial genome of *P. homarus* was 15,665 bp long and showed significant AT bias (67% AT content, 33% GC content). The A + T-rich region included copy-related control information and a poly (dT) structure that related to replication and transcription. In this study, the gene arrangement was consistent with other *Palinura* mitochondrial genomes and the sequence was strikingly similar to *Panulirus ornatus*, which would be useful in species identification and natural resources conservation.

*Palinura* belongs to clade Reptantia of Decapoda, Crustacea. Twenty-one types of *Palinura* have been uncovered; of these, the mitochondrial genomes of only *P. stimpsoni*, *P. ornatus*, and *P. japonicus* have been sequenced (Yamauchi et al. 2002; Liu & Cui 2011; Qian & Zhao 2011). The complete mitochondrial genome sequence of *P. homarus* has not yet been reported. Considering their biological diversity and a wide distribution range, *Palinura* species have drawn the attention of several evolutionary biologists. Owing to the diversity of their ecology, morphology, and behaviours, the phyletic evolution of *Palinura* has become the subject of significant controversy.

*P. homarus* was collected from offshore waters of Zhanjiang, Guangdong Province (20.580544°N, 109.737567°E) and preserved in absolute ethanol. Total genomic DNA was extracted from the tissues of the animal stored at −80 °C using the Qiagen DNEasy tissue kit (Qiagen, Hilden, Germany). Three fragments of *cox1*, *nd5* and *cytb* genes were first amplified by PCR with the universal primers (Folmer et al. 1994; Boore 1999; Lavrov et al. 2004). The conserved regions were identified by alignment of the sequences obtained from the above gene fragments with the complete mitochondrial genomes of *Panulirus stimpsoni* (Accession no. NC_014339), *Panulirus japonicas* (Accession no. NC_004251), and *Panulirus ornatus* (Accession no. NC_014854) available in GenBank. From the conserved regions derived from the aforementioned available data from GenBank, 19 pairs of PCR Primers were designed using Primer 5.0 (Palo Alto, CA) cover the entire mitochondrial genome of *P. homarus*. The primers were synthesized by Sangon Biotech Shanghai Co. Ltd (Shanghai, China). The PCR products were electrophoresed on a 1% sepharose gel (Sepharose Gel), and stained with ethidium bromide for band characterization via ultraviolet transillumination. The PCR products were purified using a Gel Extraction 50 kit (Sangon Biotech) and sent for DNA sequencing at Sangon Biotech Co., Ltd. (Shanghai, China). The gene arrangement of mitochondrial genome of *P. homarus* was identical to that of other *Palinurus*. The tRNA rearrangement was not observed. No rearrangement of the ancestral tRNA^Lys^ (K)–tRNA^Asp^ (D) arrangement has swapped to the arrangement tRNA^Asp^ (D)–tRNA^Lys^ (K). The mtDNA sequence of *P. homarus* contains 22 tRNA genes. Except for leucine (Leu) and serine (Ser), which have two corresponding tRNAs, other amino acids only have one corresponding tRNA. Through predictions of the secondary structure of the srRNA of *P. homarus*, 16S rRNA showed 6 structural domains and 53 stem-and-loop structures; 12s RNA showed 3 structural domains and 40 stem-and-loop structures. At its 3′ terminal region, a stem-and-loop structure suggested by Cannone et al. (2002), such as 12S rRNA and its similar structures, was observed. The secondary structure of the srRNA of *P. homarus* is similar to that of vertebrates (Hixson & Brown 1986), thereby demonstrating that the evolution of rRNA proceeds very slowly. Furthermore, the molecular arrangement of this srRNA showed little change, and its function was maintained over the long process of evolution.

The phylogenetic tree (Figure 1) was constructed by the maximum-likelihood methods using complete mitochondrial genomes of 17 decapoda species. The tree supports clear phylogenetic relationships at the genus level. This mitochondrial genome (*P. homarus*) represents the seventh mitochondrial genome to be published for *Panulirus*, and provides additional insight into evolutionary relationships within the genus.

**Figure 1.**
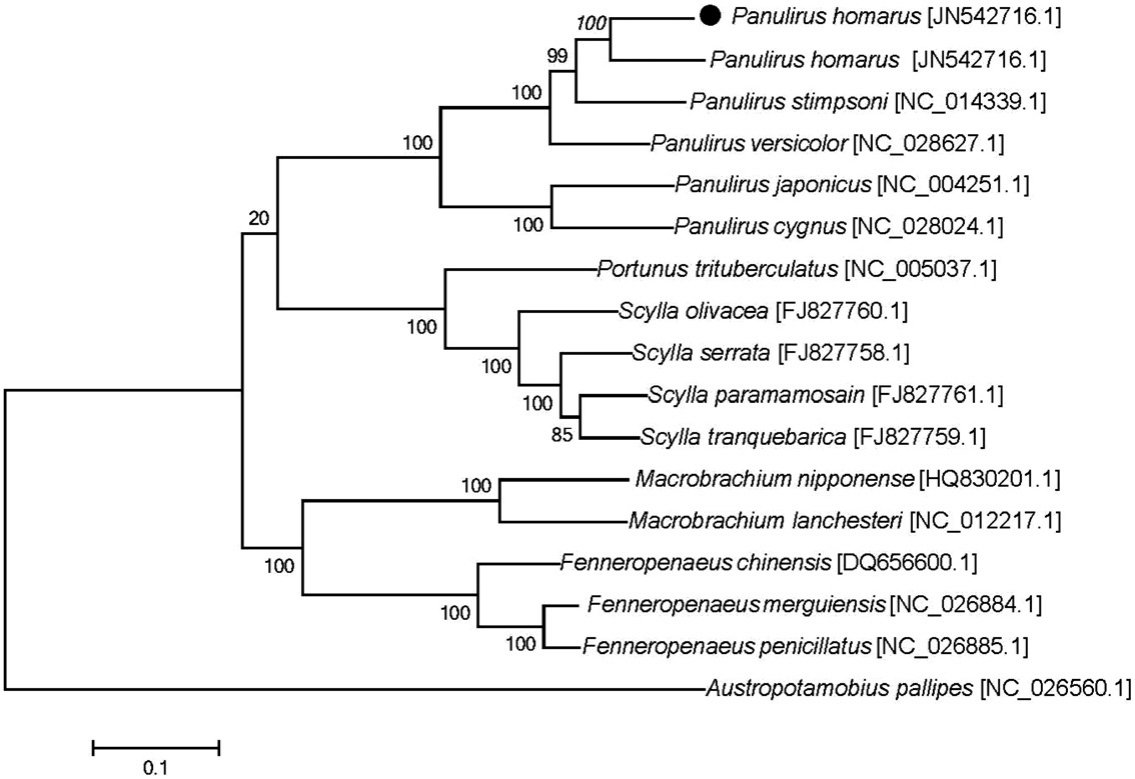
A maximum-likelihood tree constructed under the GTR model using the complete sequences of mitogenomes with *Austropotamobius pallipes* (GenBank accession number NC_026560.1) as an outgroup. The dot indicated the species in this study. Bootstrap support was shown at nodes.

## References

[CIT0001] Boore JL. 1999 Animal mitochondrial genomes. Nucleic Acids Res. 27:1767–1780. 10101183 10.1093/nar/27.8.1767 PMC148383

[CIT0002] Cannone JJ, Subramanian S, Schnare MN, Collett JR, D’Souza LM, Du Y, Feng B, Lin N, Madabusi LV, Müller KM 2002 The comparative RNA web (CRW) site: an online database of comparative sequence and structure information for ribosomal, intron, and other RNAs. BMC Bioinformatics. 3:15. 10.1186/1471-2105-3-2 PMC65690 11869452

[CIT0003] Folmer O, Black M, Hoeh W, Lutz R, Vrijenhoek R 1994 DNA primers for amplification of mitochondrial cytochrome c oxidase subunit I from diverse metazoan invertebrates. Mol Mar Biol Biotechnol. 3:294–299. 7881515

[CIT0004] Hixson JE, Brown WM. 1986 A comparison of the small ribosomal RNA genes from the mitochondrial DNA of the great apes and humans: sequence, structure, evolution, and phylogenetic implications. Mol Biol Evol. 3:1–18. 3444394 10.1093/oxfordjournals.molbev.a040379

[CIT0005] Lavrov DV, Brown WM, Boore JL, et al 2004 Phylogenetic position of the Pentastomida and (pan) crustacean relationships. Proc Royal Soc B. 271:537–544. 10.1098/rspb.2003.2631 PMC1691615 15129965

[CIT0006] Liu Y, Cui Z. 2011 Complete mitochondrial genome of the Chinese spiny lobster *Panulirus stimpsoni* (Crustacea: Decapoda): genome characterization and phylogenetic considerations. Mol Biol Rep. 38:403–410. 20352347 10.1007/s11033-010-0122-2

[CIT0008] Qian GH, Zhao Q. 2011 Two new decapod (Crustacea, Malacostraca) complete mitochondrial genomes: bearings on the phylogenetic relationships within the Decapoda. Zool J Linnean Soc. 162:471–481.

[CIT0007] Yamauchi MM, Miya MU, Nishida M 2002 Complete mitochondrial DNA sequence of the Japanese spiny lobster, *Panulirus japonicus* (Crustacea: Decapoda). Gene. 295:89–96. 12242015 10.1016/s0378-1119(02)00824-7

